# Exploring Anatomical Variations in the Bony Architecture of the Greater Palatine Canal in Dry Bones

**DOI:** 10.7759/cureus.70753

**Published:** 2024-10-03

**Authors:** Sahar Hafeez, Ali Z Ansari, Srihita Patibandla, Nilay Bhatt, Mohammed A Khan, Muhammad Malik, Abdulmanan Abid, Summaya F Khan, Laasya Patibandla

**Affiliations:** 1 Department of Pathology and Laboratory Medicine, William Carey University College of Osteopathic Medicine, Hattiesburg, USA; 2 Department of Internal Medicine, Trinity Health Grand Rapids, Grand Rapids, USA; 3 Department of Internal Medicine, Merit Health Wesley, Hattiesburg, USA; 4 Department of Internal Medicine, William Carey University College of Osteopathic Medicine, Hattiesburg, USA; 5 Department of Internal Medicine, Windsor University School of Medicine, Basseterre, KNA; 6 Department of Medical Sciences, Western University, London, CAN

**Keywords:** anatomical variations, craniomaxillofacial surgery, greater palatine canal, malfusion, nasopharynx, oral cavity, oropharynx, otolaryngology, palatine bone, skull base

## Abstract

Introduction

The greater palatine canal (GPC) holds significant clinical importance due to its role in providing access to the branches of the maxillary division of the trigeminal nerve. Anatomical variations within this posterior maxillary region can complicate the surgical anatomy, making the identification of vital structures challenging. Therefore, a thorough understanding of both normal anatomy and common anatomical variations of the GPC is essential to minimize perioperative complications during surgical procedures. This study aims to investigate the bony architecture of the GPC in dry bones to identify anatomical variations and address significant lacunae in our current understanding of this structure. Despite its impact on the management of various dental and surgical procedures, there remains a limited and sometimes inconsistent knowledge of the normal and variant anatomy of the GPC. Existing literature often lacks comprehensive detail regarding the range of anatomical variations and their implications for surgical approaches. By systematically documenting these variations, this study aims to bridge these gaps in knowledge, justify the need for continued research in this area, and highlight its potential clinical implications.

Materials and methods

In total, 30 dried and intact adult skull specimens were selected, consisting of 19 males (63.3%) and 11 females (36.7%). The selection criteria included an intact hard palate with fully erupted third molars and an intact lateral nasal wall on both sides. The presence of erupted third molars was used to assess the degree of bone resorption, ensuring that only specimens with minimal bone loss were included. The exclusion criteria ruled out specimens with major craniofacial deformities, excessive bone resorption, and signs of advanced age. The bony walls of the GPC were observed by passing a black wire made of rubber material with a consistent diameter (approximately 5 mm), allowing us to qualitatively assess the bore of the canal.

Results

In four out of 30 specimens (13.3%), significant variations were noted in the bony medial wall of the GPC. Of these, three specimens were male (75%) and one was female (25%). These variations have been categorized into four distinct types for clarity and analysis. Type 1, in a male specimen, showed a deficiency in the lower segment of the bony medial wall of the left GPC above the greater palatine foramen. Type 2, in a male specimen, had a small bar of bone in the left GPC midway between the foramen and the pterygopalatine fossa. Type 3, in a female specimen, exhibited a small bar of bone above the greater palatine foramen on the right side, with no wall above it. Type 4, in a male specimen, displayed a bar of bone above the greater palatine foramen on the right side, with the medial wall completely absent on the left side.

Conclusion

The study identified anatomical variations in the bony architecture of the GPC based on dry bone specimens. While these findings offer insights into potential variations that could affect surgical procedures, their clinical implications need validation through patient-based studies. Understanding these variations is crucial for improving preoperative planning and reducing surgical risks, but recommendations should be cautious given the limited sample size. Further research with larger samples and clinical validation is needed to fully understand the embryological basis and potential impact on surgical practice.

## Introduction

The anatomy of the greater palatine canal (GPC) is of significant interest to dentists, oral and maxillofacial surgeons, and otolaryngologists performing various procedures in this region, such as local anesthesia administration, dental implant placement, orthognathic Le Fort I osteotomies, and sinonasal surgeries [1]. The GPC begins at the tip of the pterygopalatine fossa and terminates at the summit of the greater palatine foramen on the posterior surface of the hard palate, formed by the horizontal plate of the palatine bone [2]. It descends inferiorly from the pterygopalatine fossa tip, traversing within the posterior-most part of the bony lateral wall of the nose, thereby closely associated with the nasopharynx [3]. The average length of the canal is approximately 28-30 mm in males and 27-29 mm in females [4]. Within this canal, the descending palatine artery (a branch of the third division of the maxillary artery) and the greater and lesser palatine nerves (branches of the maxillary division of the trigeminal nerve) are housed, along with their posterior inferior lateral nasal branches [5].

As the GPC transmits neurovascular structures supplying the hard and soft palate and the posterior part of the nasal cavity, it is crucial to understand the bony architecture and important relations of this canal [6]. As described classically, the GPC is formed by the apposition of an obliquely descending groove on the posteroinferior aspect of the maxilla and an elongated groove on the lateral surface of the perpendicular plate of palatine bone [7]. Therefore, we can say that the bony medial wall of the GPC is formed by the fused portion of the posterior maxilla and the perpendicular plate of the palatine bone, while the lateral wall of the canal is formed by the pterygomaxillary fissure [8]. The maxillary artery and the posterior superior alveolar nerve traverse the pterygomaxillary fissure, extending from the infratemporal fossa into the pterygopalatine fossa. Additionally, in Le Fort I osteotomy, a procedure commonly performed in orthognathic surgery, unfavorable fractures of the pterygomaxillary disjunction may occur, often accompanied by injury to the descending palatine artery [9].

During life, a thick mucoperiosteum covers the entire length of the medial bony wall of the GPC on the nasal side [10]. The maxillary sinus is anterior, the nasal cavity and conchae are medial, and the pterygoid process is posterior to the GPC. The maxillary hiatus is posteriorly closed by the perpendicular plate of the palatine bone and is typically located within the third segment of the posteroinferior infundibulum [11]. Any anatomical disfigurement of these structures undoubtedly affects the anatomy of the GPC due to their proximal relationships [12]. Preservation of the descending palatine artery and palatine nerves is essential when performing surgical procedures in this area to avoid excessive bleeding and maintain nerve supply to the maxilla [13]. While numerous studies have examined the length and direction of the GPC, limited attention has been given to its bony architecture. In this study, we aim to investigate the osseous anatomy of the GPC and identify variations in its bony structure.

## Materials and methods

Specimen selection and exclusion criteria

A total of 40 dried and intact adult skull specimens were initially examined from the skull collection of the Department of Clinical Anatomy at Western University in Ontario, of which 30 (n = 30, 19 males (63.3%) and 11 females (36.7%)) were selected based on the inclusion criteria. Ten skulls were excluded as they did not meet these criteria. The criteria included an intact hard palate with fully erupted third molars and an intact lateral nasal wall on both sides. The presence of erupted third molars was specifically used as an indicator to assess the degree of bone resorption, ensuring that only specimens with minimal bone loss were included. Specimens were excluded if they exhibited major craniofacial deformities (e.g., cleft palate), excessive bone resorption, or signs of advanced age that could compromise the integrity of the anatomical structures. The study was limited to 30 skulls due to the availability of specimens meeting these criteria and the need for a homogeneous sample to ensure reliable measurements.

Examination procedure

A detailed examination of the posterior maxillary region of each specimen was conducted independently by five authors (SH, AZA, SP, NB, and MAK), all with advanced training in anatomy. The bony architecture of the pterygopalatine fossa, pterygomaxillary fissure, and GPC was meticulously examined to identify any malformations or deviations from standard anatomical features. To ensure inter-rater reliability, all measurements and observations were cross-verified by the five authors, with any discrepancies resolved through consensus discussions.

Use of black rubber wire for GPC observation

To facilitate the observation of the bony walls of the GPC, a black wire made of rubber material with a consistent diameter of approximately 5 mm was carefully introduced through the canal in each specimen. The rubber wire was chosen for its flexibility and ability to conform to the shape of the canal, allowing for a qualitative assessment of the canal's bore and ensuring accurate identification of its anatomical boundaries. The primary purpose of using the wire was to enhance the contrast against the bone, making it easier to visualize and assess any variations or malformations. No other purposes for the wire's use were intended.

Definition of standard anatomy

**Figure 1:**
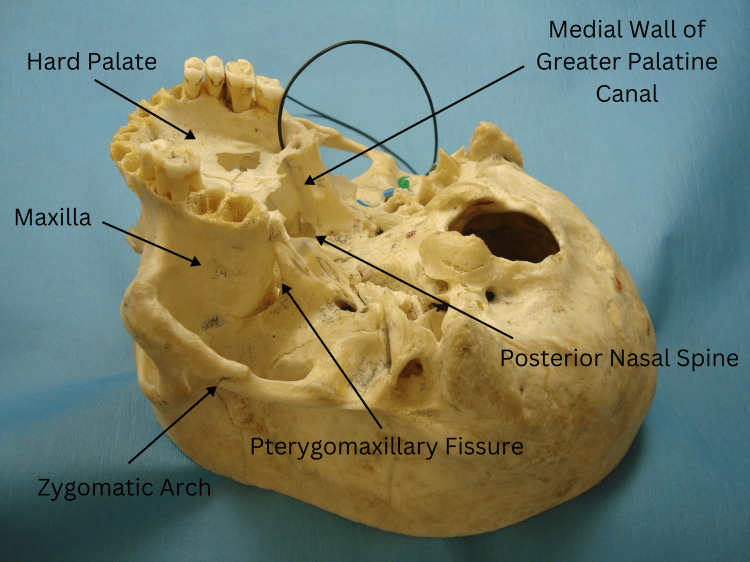
The black rubber wire inserted into the greater palatine foramen passes through the left GPC. Note that the wire is not visible from the nasal side due to the intact bony medial wall. GPC: greater palatine canal

The standard anatomy of the GPC was defined based on established anatomical references, including numeric values for the canal's length, the angle between the hard palate and the canal, and the canal's diameter (Figure 1). These parameters were compared against our observations to identify any deviations.

Ethics and institutional approval

This study did not involve live human participants or animals, but it was conducted in accordance with institutional guidelines for research on human remains. Ethical approval for the use of human skeletal material was granted by the Research Ethics Board of Western University in Ontario. All specimens were part of the pre-existing skull collection of the Department of Clinical Anatomy at Western University. Informed consent was not applicable as the specimens were anonymized and obtained through legal and ethical channels.

## Results

In this study involving 30 specimens, significant anatomical variations were identified in the bony medial wall of the GPC in four specimens, representing 13.3% of the total (p < 0.05). These statistically significant variations indicate deviations from the expected normal anatomy. The variations were classified into four distinct types.

**Figure 2:**
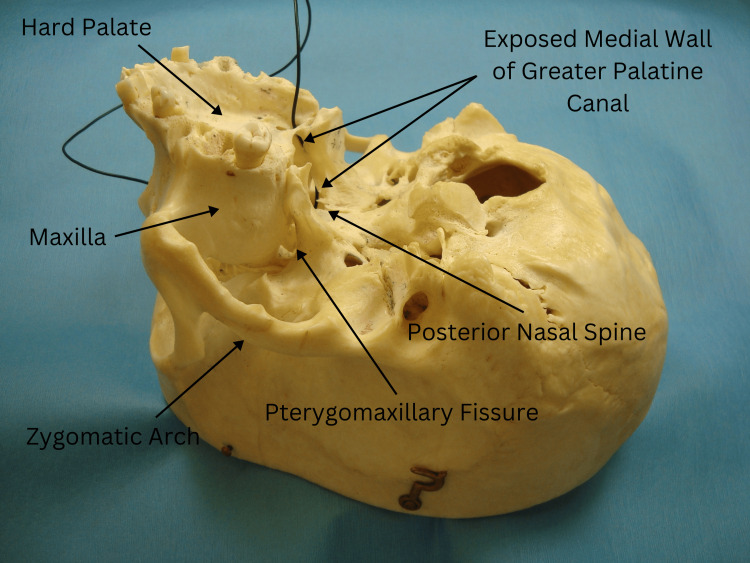
Type 1 variation in a male specimen showing a deficiency in the bony medial wall of the left GPC above the greater palatine foramen, with a black wire visibly passing through from the nasal cavity. GPC: greater palatine canal

Type 1, observed in a male specimen, displayed a deficiency in the lower segment of the bony medial wall of the left GPC above the greater palatine foramen. This finding was evident in Figure 2, where the intact right GPC contrasted with the left side, allowing a black wire to pass visibly through the canal from the nasal cavity.

**Figure 3:**
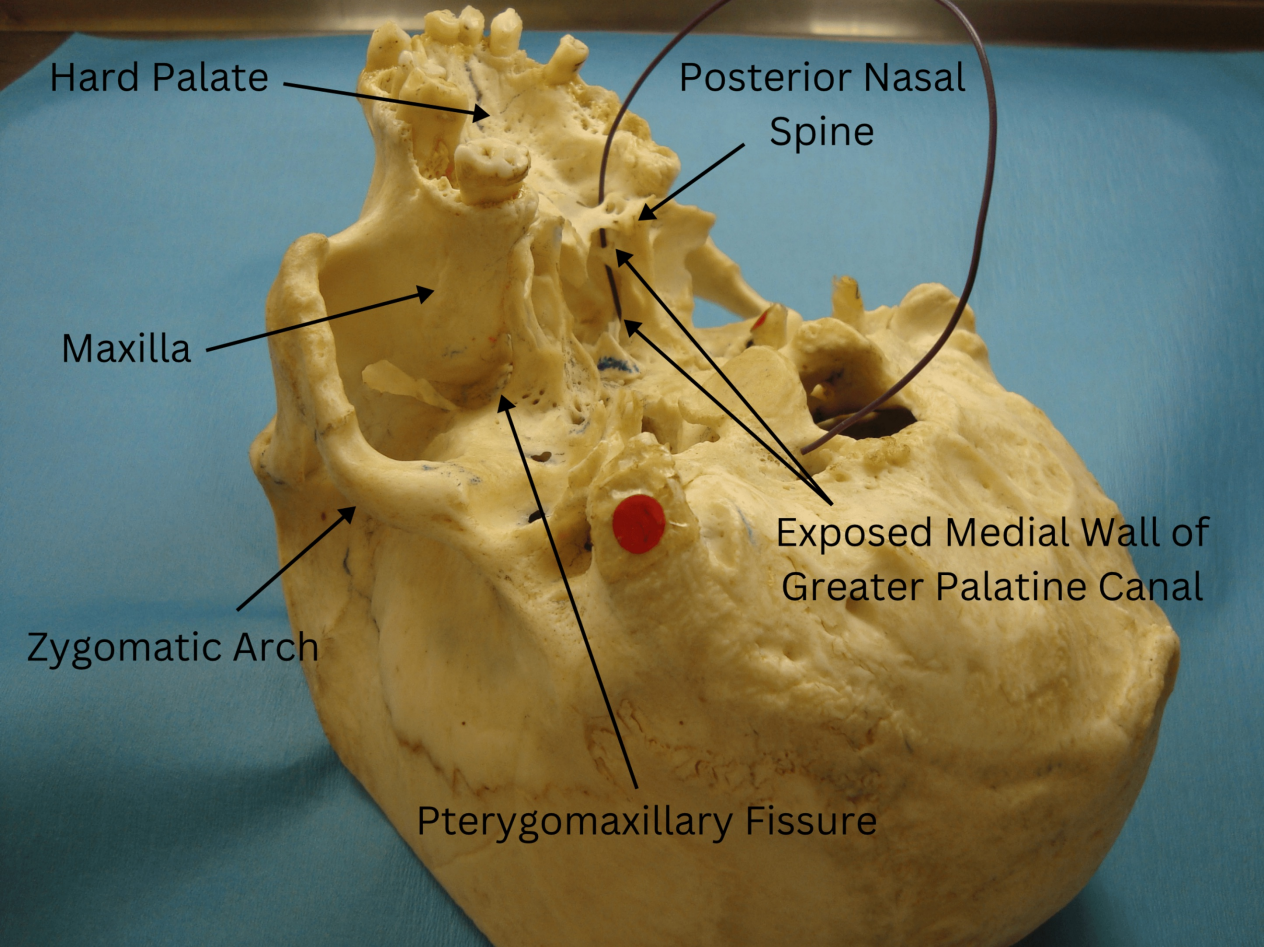
Type 2 variation in a male specimen showing a small bar of bone in the left GPC midway between the greater palatine foramen and the tip of the pterygopalatine fossa, with a black wire visible from the nasal cavity, passing both above and below the bar. GPC: greater palatine canal

Type 2, also in a male specimen, revealed a small bar of bone in the left GPC located midway between the greater palatine foramen and the tip of the pterygopalatine fossa (Figure 3). The black wire was similarly visible from the nasal cavity, traversing both above and below this bony bar.

**Figure 4:**
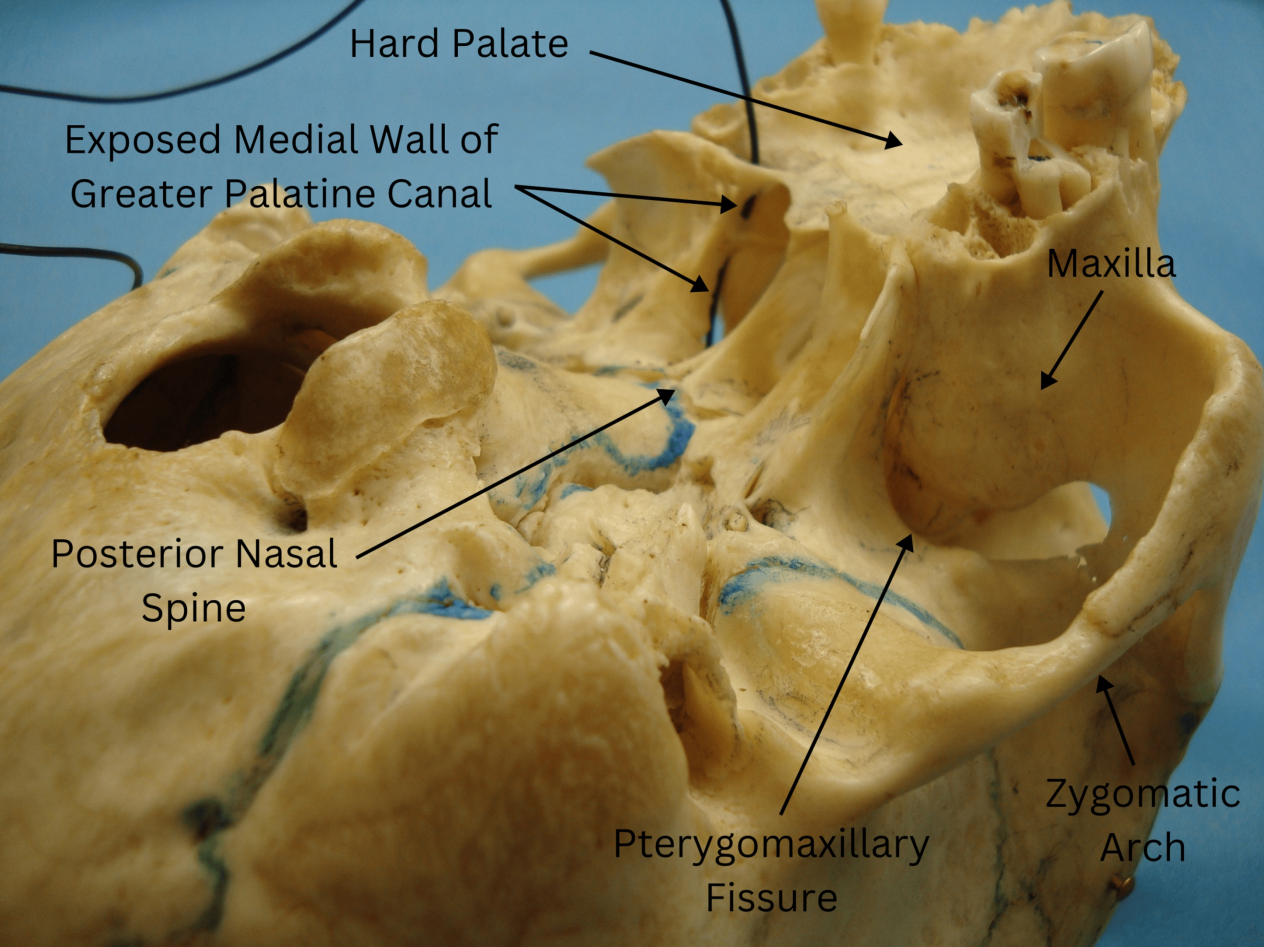
Type 3 variation in a female specimen showing a small bar of bone just above the greater palatine foramen on the right side with no bony wall above it, with a black wire passing both above and below the bar, while the left side's medial bony wall remains intact.

Type 3, identified in a female specimen (Figure 4), showed a small bar of bone just above the greater palatine foramen on the right side, with no bony wall present above it. The black wire could be seen passing above and below this bar, while the left side maintained an intact medial bony wall.

**Figure 5:**
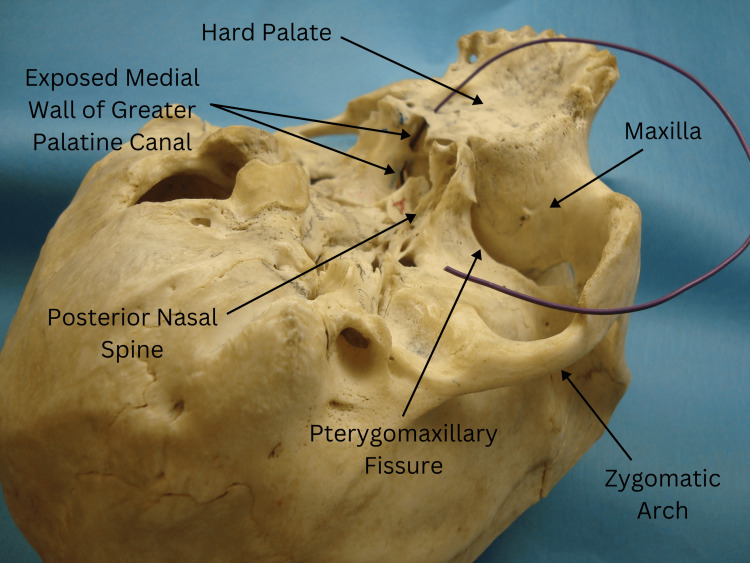
Type 4 variation in a male specimen showing a bar of bone positioned 2 mm above the greater palatine foramen on the right side, with a black wire visible from the nasal cavity between the greater palatine foramen and the bar.

**Figure 6:**
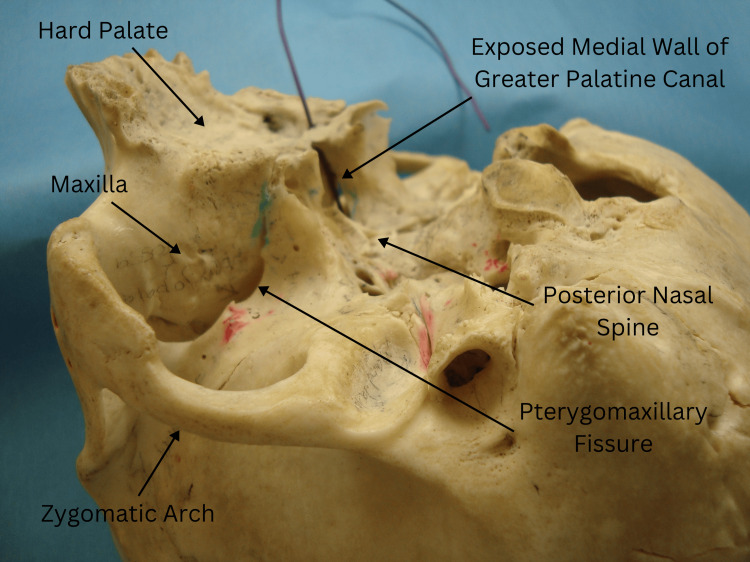
Type 4 variation in the same male specimen showing the absence of the medial wall on the left side, with a black wire fully visible from the nasal cavity along the entire length of the GPC. GPC: greater palatine canal

Finally, type 4, found in a male specimen, exhibited a bar of bone positioned 2 mm above the greater palatine foramen on the right side (Figure 5), with the medial wall absent on the left (Figure 6). In this case, the black wire was fully visible from the nasal cavity along the entire length of the GPC, positioned between the greater palatine foramen and the bar of bone.

A summary of these findings is presented in Table 1.

**Table 1:** Summary of anatomical variations in the bony medial wall of the GPC observed in four out of 30 specimens. Each variation is categorized by type and sex, with a detailed description of the anomaly and associated figure references. GPC: greater palatine canal

Type	Sex	Description of variation	Figure
Type 1	Male	Deficiency in the lower segment of the bony medial wall of the left GPC above the greater palatine foramen; black wire visible from the nasal cavity	Figure 2
Type 2	Male	Small bar of bone in the left GPC midway between the greater palatine foramen and the pterygopalatine fossa; black wire visible both above and below the bar	Figure 3
Type 3	Female	Small bar of bone observed just above the greater palatine foramen on the right side; no bony wall above it; black wire visible passing above and below the bar	Figure 4
Type 4	Male	Bar of bone 2 mm above the greater palatine foramen on the right side; medial wall absent on the left side; black wire fully visible from the nasal cavity	Figure 5 and Figure 6

## Discussion

Our findings highlight novel variations in the bony medial wall of the GPC, which may influence clinical approaches to this area. We identified anatomical variations in four out of 30 dried skull specimens, representing 13.3% of the sample. This discovery adds valuable insight to the current body of knowledge, as previous research on the GPC has rarely emphasized the presence of these bony anomalies. The variations observed in our study contribute to the understanding of how such deviations from normal anatomy may pose challenges during surgical interventions, particularly in maintaining hemostasis and avoiding nerve damage during procedures [14].

Comparing our findings to prior studies, it is evident that anatomical variations in the GPC have not been extensively documented. In a study by Aşantoğrol et al., similar anatomical variations in the maxillofacial region were noted, although their focus did not include the GPC specifically [15]. Another study by Chrcanovic et al. documented variable patterns of the palatine bone but did not highlight variations in the medial wall of the GPC, further illustrating the novelty of our findings [16]. Our study bridges this gap and expands the literature by categorizing the observed variations into distinct types based on their location and structure.

Embryologically, these variations may be explained by disruptions during the ossification process of the palatine bone. As previously mentioned, the palatine bone develops through intramembranous ossification with four independent ossification centers: the perpendicular plate, orbital process, sphenoidal process, and horizontal plate. Any interruption or malformation in the ossification of the perpendicular plate may result in incomplete fusion with the maxilla, which forms the medial wall of the GPC. This hypothesis aligns with our findings, where variations such as partial or complete absence of the bony medial wall could be attributed to developmental anomalies in the fusion process during prenatal growth.

The surgical anatomy of the GPC can be further complicated by anatomical variations, making identification of vital structures challenging, particularly when intraoperative bleeding obscures the region [17]. Therefore, accurate knowledge of normal anatomy and common anatomical variations is crucial for minimizing intraoperative and postoperative complications associated with invasive microsurgical approaches to this region. The descending palatine artery, situated within the GPC, is prone to injury during osteotomy of the medial or lateral maxillary sinus walls [6]. Literature suggests that in cases where the bony wall of the canal is deficient, a segment of the artery becomes highly susceptible to surgical trauma, particularly during procedures involving the maxillary sinus [18,19]. In instances where there is a partial or complete absence of the osseous wall, this segment becomes highly susceptible to surgical trauma.

One of the major branches of the sphenopalatine artery, known as the posterior lateral nasal artery, courses downward over the perpendicular plate of the palatine bone [20]. This artery serves as the primary source of blood supply to the lateral wall of the nose [21]. In a greater palatine nerve block procedure, there is a risk of inadvertently piercing this artery with a needle if the osseous medial wall of the canal is partially or completely absent [22]. Studies have noted that in the absence of a complete osseous wall of the canal, there is an increased risk of spreading infectious or neoplastic processes through the nasal cavity or the nasopharynx [23]. Such spread may result in perineural invasion of palatine nerves or vascular seeding of the descending palatine artery.

Our findings contribute to the growing recognition of anatomical diversity in craniofacial structures and highlight the need for further investigation into the embryological factors that contribute to these variations. A more comprehensive understanding of the developmental processes that lead to these anomalies could inform not only surgical practice but also educational programs aimed at training surgeons to anticipate and manage such variations. By expanding the body of knowledge surrounding the GPC and its variations, future research can help refine surgical techniques and improve patient outcomes, ultimately advancing the field of craniofacial surgery.

## Conclusions

The present study highlights the presence of anatomical variations in the bony architecture of the greater palatine canal, which could play a critical role in surgical planning for procedures in the posterior maxillary region. Recognizing these variations is essential for minimizing surgical complications, particularly when dealing with vital vascular and neural structures in this area. Our findings emphasize the potential for bony deficiencies or bars in the canal, which may impact surgical access and increase the risk of trauma to critical structures such as the descending palatine artery. Additionally, this study suggests that some of these variations may stem from interruptions in the prenatal ossification process of the perpendicular plate of the palatine bone. To further establish the embryological basis of these findings, larger-scale studies involving more specimens and clinical validation are needed. Such research would provide a deeper understanding of the prevalence and implications of these anatomical variations, ultimately improving surgical outcomes and reducing complications in maxillofacial and related procedures.
